# Survivin and angiotensin-converting enzyme polymorphisms with risk of colorectal cancer: a systematic review and meta-analysis

**DOI:** 10.1186/s12957-015-0461-5

**Published:** 2015-02-07

**Authors:** Xile Zhou, Caizhao Lin

**Affiliations:** Department of Colorectal Surgery, The First Affiliated Hospital, College of Medicine, Zhejiang University, 79 Qingchun Road, Hangzhou, Zhejiang 310003 P.R. China

**Keywords:** Colorectal cancer, Survivin, Angiotensin-converting enzyme, Polymorphism, Meta-analysis

## Abstract

**Background:**

Colorectal cancer (CRC) is the most common cause of cancer death worldwide. Numerous studies have identified the roles of survivin −31 G/C and angiotensin-converting enzyme insertion/deletion (ACE I/D) polymorphisms in CRC risk; however, the results remain inconclusive. This study was to investigate associations between these two polymorphisms and CRC susceptibility.

**Methods:**

A comprehensive literature search was conducted to collect relevant case–control studies published between 2000 and 2014. The extracted data were statistically analyzed, and the odds ratios (ORs) with 95% confidence intervals (CIs) were employed to estimate the strength of association.

**Results:**

A total of 11 studies were included in the meta-analysis. For survivin G/C polymorphism, six articles reported 1,840 cases and 1,804 controls. Overall, we found the frequency of C allele is higher in CRC cases than that in the healthy controls (57.2% vs. 48.0%), and C allele significantly increased the risk of CRC compared to G allele in allele model (OR = 1.46, 95% CI = 1.33–1.60, *P* < 0.00001). This association was also found in other genetic models (*P* < 0.00001). Stratified analysis by ethnicity showed significant association in each genetic model among the Asian population. For ACE I/D polymorphism, five studies included 758 cases and 6,755 controls. No significant association was found in any genetic models.

**Conclusions:**

Our results showed that survivin −31 G/C polymorphism might contribute to risk of CRC, especially in the Asian populations. However, the ACE I/D polymorphism is not a genetic factor concerning the risk for CRC. More studies with larger sample sizes are required in the future.

## Background

Colorectal cancer (CRC) is one of the three most common cancers in the world and is a major contributor to cancer-related death [1]. Each year, a global incidence exceeding 1.2 million new cases emerge and 600,000 deaths occur [2]. Its incidence rates continue to increase in economically transitioning countries. According to colorectal cancer statistics, an estimated 71,830 men and 65,000 women will be diagnosed with CRC, and 26,270 men and 24,040 women will die of this disease in 2014 [3]. The etiological factors and pathogenetic mechanisms underlying CRC development appear to be complex and heterogeneous. In the last two decades, studies have demonstrated that CRC cells undergo major epigenetic alterations [4]. Among which, genetic variants in oncogenes have been extensively investigated as the essential role in cancer etiology [5].

The survivin gene, located in chromosome 17q25, is the smallest member of the inhibitor of apoptosis (IAP) gene family [6]. It is a multifunctional protein and is required to preserve tissue or organism viability [7]. Survivin is expressed in many human cancers and involved in the regulation of cell division and survival [8]. The survivin −31 G/C polymorphism may modulate susceptibility to cancer by influencing the expression of survivin. A recent study has supported a role of survivin in colorectal carcinogenesis, while the −31 G/C polymorphism may constitute a marker of survival [9].

The angiotensin-converting enzyme (ACE), a major component of the renin-angiotensin system (RAS), plays a crucial role in the regulation of circulatory homeostasis such as blood pressure and serum electrolytes [10]. It is located on human chromosome 17q23 and has been involved in the pathogenesis of human cancers [11,12]. Epidemiologic studies have indicated that inhibition of ACE activity could suppress tumor growth and angiogenesis, decreasing the risk and mortality rate [13]. Recent research showed that long-term/high-dose exposure to ACE inhibitor may decrease the incidence of CRC [14]. ACE variants might affect its activity. Among which, ACE I/D (rs4646994) polymorphism in intron 16 of this gene, based on insertion (I) or deletion (D) of a 287-bp Alu sequence, was the most widely studied and leaded to a change in the plasma ACE level. The carriers of D allele were shown in higher ACE activity.

Numerous studies have shown the association of these genetic polymorphisms with CRC [9,15-25]. However, the results remained inconsistent. In the present study, we performed meta-analyses to evaluate and summarize the contribution of the two polymorphisms to CRC susceptibility in different populations.

## Methods

### Literature search

A comprehensive literature search was conducted using the online electronic database of Embase, Medline, PubMed, CNKI (China National Knowledge Infrastructure), and Wanfang. We retrieved the relevant articles published between January 2000 and March 2014 using the following terms: “colorectal cancer or colorectal carcinoma”, “angiotensin-converting enzyme or ACE”, “survivin”, and “polymorphism or variant or mutation” as well as their combinations. The references of retrieved articles were searched manually. When the same authors or laboratory reported the issue on the same group of people, only full-text articles of the most recent studies were included.

### Study selection

The inclusion criteria were as follows: 1) case–control or cohort studies, 2) evaluating the contribution of survivin −31 G/C and ACE I/D polymorphisms with CRC risk, 3) the results presented in odds ratio (OR) with its 95% confidence interval (CI), and 4) genotype distributions in the cases and controls were available to extract.

### Data extraction

Two investigators independently assessed the data from the included studies. Any disagreement was discussed with a third expert to reach a final consensus. The following information was extracted: the name of first author, publication year, country, ethnicity, sample size, genotyping method, and the genotype frequencies in the cancer cases and controls.

### Statistical analysis

The overall effect was measured by ORs with its 95% CI. The *Z* test was employed to determine the significance of the pooled ORs, and a *P* value less than 0.05 was considered statistically significant. The per-allele model (C vs. G for survivin, D vs. I for ACE), dominant model (GC + CC vs. GG for survivin, ID + DD vs. II for ACE), recessive model (CC vs. GC + GG survivin, DD vs. ID + II for ACE), and additive model (CC vs. GG for survivin, DD vs. II for ACE) were examined to assess these association. The *I*^2^ test and the *Q* statistic test were used to assess the between-study heterogeneity. The Mantel-Haenszel (M-H) fixed-effects model is used when the *P* value is more than 0.10 for the *Q* test and less than 50% for *I*^2^; otherwise, the random-effects model is used. The publication bias was assessed by visual funnel plot inspection. Review Manager (version 5.2, The Cochrane Collaboration) was used to conduct the statistical analyses. All the tests were two sided.

## Results

### Study selection and characteristics

**Figure 1:**
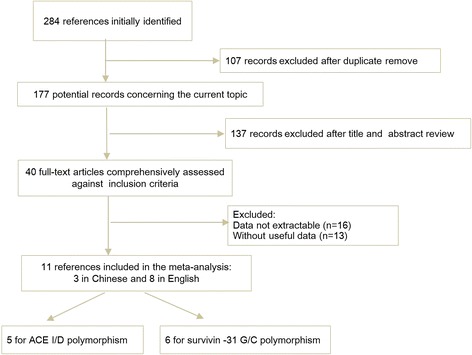
**Flow chart of literature screening.**

The initial search identified 284 references. Of those, 107 records were excluded for duplication and 177 articles were judged potentially relevant. Following the title and abstract screening, 40 full-text articles met inclusion criteria. Overall, a total of 11 studies (eight in English and three in Chinese) were finally included in this review. Figure 1 showed the study flow.

Of the 11 case–control studies, there were five studies of Asians and six of Caucasian. For ACE I/D polymorphism, five studies containing 758 cases and 6,755 controls were included. For survivin G/C polymorphism, six articles reported 1,840 cases and 1,804 controls. Table 1 summarized the main characteristics of included studies. Table 2 showed the information of genotypes and alleles for each case–control study.

**Table 1:** **Main characteristic of the eligible studies**

**First author**	**Year**	**Country**	**Ethnicity**	**Cases**	**Controls**	**Genotyping method**
Survivin (−31 G/C)						
Gazouli [16]	2009	Greece	Caucasian	312	362	PCR-RFLP
Huang [17]	2010	China	Asian	702	711	PCR-RFLP
Antonacopoulou [9]	2011	Greece	Caucasian	163	132	Taqman
Duan [18]	2012	China	Asian	206	129	PCR-RFLP
Liu [19]	2012	China	Asian	182	200	PCR-LDR
Li [20]	2013	China	Asian	275	270	PCR-RFLP
ACE (I/D)						
Nikiteas [21]	2007	Greece	Caucasian	92	102	PCR
Rocken [22]	2007	Germany	Caucasian	141	189	PCR
Van der [23]	2008	Netherlands	Caucasian	176	6,015	PCR
Toma [24]	2008	Romanian	Caucasian	108	150	PCR
Liu SY [25]	2011	China	Asian	241	299	PCR-PAGE

**Table 2:** **Distribution of genotypes in the individual studies**

	**Cases**					**Control**				
Survivin	GG	GC	CC	G	C	GG	GC	CC	G	C
Gazouli [16]	68	131	113	267	357	123	163	76	409	315
Huang [17]	144	302	256	590	814	180	345	186	705	717
Antonacopoulou [9]	63	84	16	210	116	66	50	16	182	82
Duan [18]	31	92	83	154	258	32	66	31	130	128
Liu [19]	36	76	70	148	216	54	93	53	201	199
Li [20]	42	123	110	207	343	55	138	77	248	292
ACE	II	ID	DD	I	D	II	ID	DD	I	D
Nikiteas [21]	15	27	50	57	127	6	44	52	56	148
Rocken [22]	37	69	35	143	139	41	95	53	177	201
Van derv [23]	34	97	45	165	187	1,332	3,006	1,677	5,670	6,360
Toma [24]	25	50	33	100	116	30	73	47	133	167
Liu SY [25]	71	138	32	280	202	95	158	46	348	250

### Association of survivin G/C polymorphism and CRC risk

**Figure 2:**
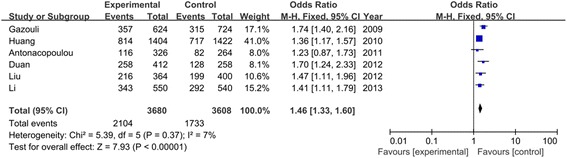
**Association between C allele of survivin −31 G**/**C polymorphism and CRC risk.**

**Figure 3:**
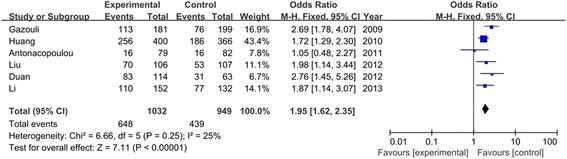
**Forest plot of odd ratios** **(ORs)** **of survivin polymorphism** **(CC vs. GG)** **associated with CRC risk.**

**Figure 4:**
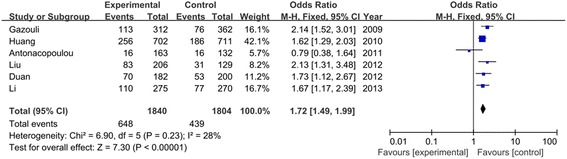
**Meta** **-analysis of survivin polymorphism** **(CC vs. GC +** **GG)** **with risk of CRC.**

**Figure 5:**
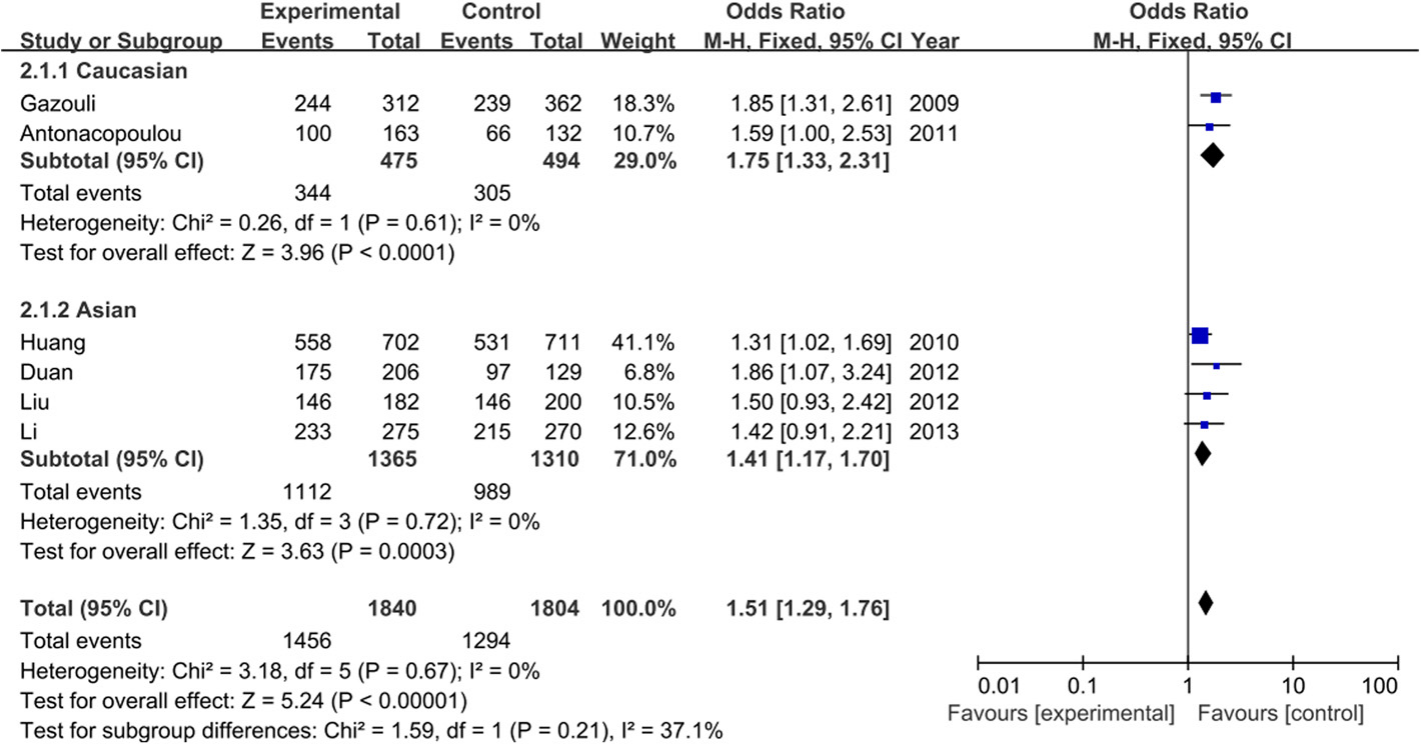
**Forest plot of CRC associated with distribution of survivin polymorphism** **(CC +** **GC vs. GG)** **by stratified analysis.**

Table 3 listed the results of allele and genotypes of survivin polymorphism in this meta-analysis. The between-study heterogeneity was not significant and the fixed-effect model was employed. Overall, the frequency of C allele was higher in CRC cases than that in the healthy controls (57.2% vs. 48.0%). As shown in Figure 2, we found that C allele significantly increased the risk of CRC compared to G allele in the allele model (OR = 1.46, 95% CI = 1.33–1.60, *P* < 0.00001). This significant association was also found in other genetic models (CC vs. GG: OR = 1.95, 95% CI = 1.62–2.35, *P* < 0.00001; CC + GC vs. GG: OR = 1.51, 95% CI = 1.29–1.76, *P* < 0.00001; CC vs. GC + GG: OR = 1.72, 95% CI = 1.49–1.99, *P* < 0.00001) as shown in Figures 3 and 4. When evaluating the effect of the polymorphism by ethnicity, we found a significant association in all genetic models among the Asian population (C vs. G: OR = 1.42, 95% CI = 1.27–1.58, *P* < 0.00001; CC vs. GG: OR = 1.88, 95% CI = 1.52–2.33, *P* < 0.00001; CC + GC vs. GG: OR = 1.41, 95% CI = 1.17–1.70, *P* = 0.0003; CC vs. GC + GG: OR = 1.70, 95% CI = 1.44–2.01, *P* < 0.00001) in a fixed-effect model. Among the Caucasian population, Figure 5 showed that C allele carrier was significantly associated with the increased risk of CRC compared to healthy control groups (CC + GC vs. GG: OR = 1.75, 95% CI = 1.33–2.31, *P* < 0.0001). No relationship was found in other genetic models (*P* > 0.05).

**Table 3:** **Meta**-**analysis of survivin −31 G**/**C polymorphism in CRC by ethnicity analysis**

	**Total**			**Asian**			**Caucasian**		
	**OR (95% CI)**	***P***	**Ph**	**OR (95% CI)**	***P***	**Ph**	**OR (95% CI)**	***P***	**Ph**
C vs. G	1.46 (1.33, 1.60)	<0.00001	0.37/7%	1.42 (1.27, 1.58)	<0.00001	0.64/0%	1.50 (1.07, 2.10)	0.02	0.09/64%
CC vs. GG	1.95 (1.62, 2.35)	<0.00001	0.25/25%	1.88 (1.52, 2.33)	<0.00001	0.62/0%	1.78 (0.71, 4.45)	0.22	0.04/77%
CC + GC vs. GG	1.51 (1.29, 1.76)	<0.00001	0.67/0%	1.41 (1.17, 1.70)	0.0003	0.72/0%	1.75 (1.33, 2.31)	<0.0001	0.61/0%
CC vs. GC + GG	1.72 (1.49, 1.99)	<0.00001	0.23/28%	1.70 (1.44, 2.01)	<0.00001	0.80/0%	1.37 (0.52, 3.62)	0.52	0.02/83%

### Association of ACE I/D polymorphism and CRC risk

**Figure 6:**
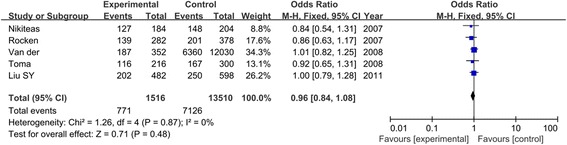
**Association between D allele of ACE I** **/D polymorphism and CRC risk.**

Table 4 displayed the summary of all genetic comparisons between ACE I/D polymorphism and CRC risk. The *I*^2^ was less than 50% and the *P* value was greater than 0.01, suggesting no heterogeneity was present. As shown in Figure 6, the result suggested that the variant D allele did not have a significant increased risk of CRC compared with those individuals without D allele (D vs. I: OR = 0.96, 95% CI = 0.84–1.08, *P* = 0.48). No significant association was found in other genetic models (DD vs. II: OR = 0.86, 95% CI = 0.66–1.12, *P* = 0.25; DD + ID vs. II: OR = 0.97, 95% CI = 0.79–1.19, *P* = 0.77; DD vs. ID + II: OR = 0.91, 95% CI = 0.74–1.12, *P* = 0.39).

**Table 4:** **Meta**-**analysis of ACE I**/**D polymorphism in CRC**

**Genotype**	**OR** **(95%** **CI)**	***P***	**Ph**	***I*** ^**2**^ **(%)**	**Model**
D vs. I	0.96 (0.84, 1.08)	0.48	0.87	0	Fixed
DD vs. II	0.86 (0.66, 1.12)	0.25	0.48	0	Fixed
DD + ID vs. II	0.97 (0.79, 1.19)	0.77	0.12	46	Fixed
DD vs. ID + II	0.91 (0.74, 1.12)	0.39	0.93	0	Fixed

### Sensitivity analysis and publication bias

**Figure 7:**
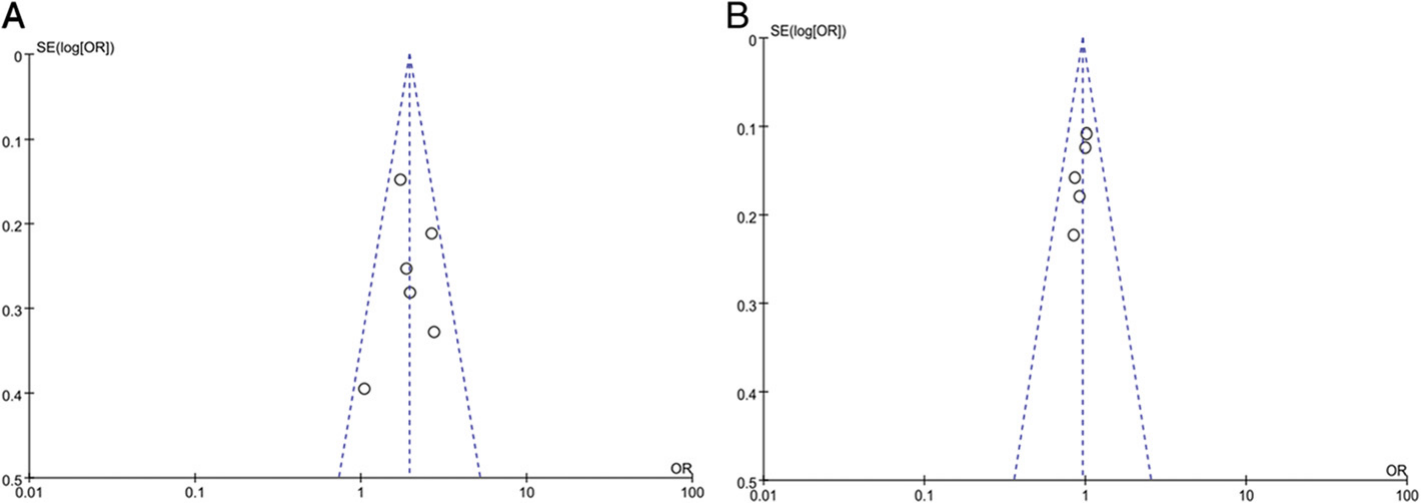
**Begg’** **s funnel plot for publication bias in selection of studies (A for survivin,** **B for ACE).**

A single study included in the meta-analysis was deleted each time to reflect the influence of the individual data set to the pooled ORs. The corresponding pooled ORs were not materially changed, which confirmed the stability of our overall result. The shape of funnel plots did not reveal any evidence of funnel plot asymmetry (Figure 7).

## Discussion

CRC is a major cause of morbidity and mortality worldwide. Although surgical resection remains a curative option for CRC treatment, most patients with CRC are not suited for surgical operations because of the advanced stage of the lesions when diagnosed. Thus, there is an urgent need to identify newer biomarkers to evaluate the severity of this disease, predict the prognosis of patients, and target the pathogenic genes.

Recently, survivin has been reported to be overexpressed in almost all human tumors. It is considered as a tumor-associated antigen for immune-based clinical approaches in CRC [26]. Researchers have suggested that survivin is implicated in the progression of dysplasia to neoplasia in the colon [27]. A significant role of survivin in CRC progression and recurrence of colon cancer liver metastases has been previously demonstrated [28]. The mechanisms of the survivin re-expression in malignancies are still unclear due to the complexity of its regulation. Genetic variations located in the promoter of the survivin gene have impact on the protein expression and consequently contributed to the genetic susceptibility to cancers. The −31 G/C polymorphism is the most extensively studied variant located in the promoter region of survivin, and the C allele has been identified to be associated with a higher transcription activity of survivin [29]. This meta-analysis showed that the survivin −31 G/C polymorphism was associated with increased CRC risk in total populations as well as in Asians, while no association was found in Caucasian except the dominant model. Our result is in agreement with the study conducted by Qin et al., which identified that this polymorphism is associated with elevated cancer risk [30].

Survivin acts as an antagonist of apoptotic cell death and functions as a regulator of mitosis [31,32]. It is a potentially interesting prognostic marker, implicating a shift from adenoma with low dysplasia to high dysplasia during human colorectal tumorigenesis [27]. Survivin overexpression was related with the increase of invasion and the metastasis of CRC [33]. A study conducted by Choi et al. suggested that the nuclear expression of survivin might be associated with the metastasis of CRC to the liver [34]. Survivin can be regulated by or cooperated with other genes. The stimulation of survivin expression by TCF/β catenin might contribute to the molecular pathogenesis of CRC [35]. The positive expression of survivin protein, directly correlated to that of Bcl-2, acted on different stages of apoptosis to promote jointly the development of CRC in a synergistic way [36]. The combined expression levels of Aldh1, survivin, and EpCAM as strong independent prognostic factors for survival and tumor recurrence in colon cancer patients reflect tumor aggressiveness [37]. Furthermore, survivin is also a therapeutic target in cancers [38]. Survivin expression increases during the normal mucosa-adenoma-carcinoma sequence and is maintained throughout the progression of disease, which strengthens its appeal as a therapeutic target [39]. The expression of survivin may play a role in identifying a subgroup of patients who could benefit from a targeted therapy against survivin in CRC [40].

Much evidence indicates that ACE associated with the pathology of carcinomas. It is differentially expressed in several malignancies. ACE is a monomeric glycoprotein that is distributed in many tissues and biological fluids [41]. ACE can influence tumor cell migration, proliferation, metastatic behavior, and angiogenesis [42]. The expression of ACE is upregulated in several cancers with functions of angiogenesis and tumor cell growth [43].

Previous meta-analysis showed that the ACE I/D polymorphism is associated with hepatocellular carcinoma (HCC), indicating that this polymorphism contributes to HCC progression in the Chinese population [44]. Recent analysis suggested that the ACE I/D polymorphism might not be a common risk factor for overall cancer susceptibility [43]. Our results did not show any association between ACE I/D polymorphism and CRC in each genetic models (*P* > 0.05). This is in accordance with meta-analysis conducted by Liu et al. and Zhang et al. [12,45].

Several limitations were presented in this meta-analysis. Firstly, the number of included studies in the subgroup analysis was small which may have a relatively lower power. Secondly, other covariates such as age, sex, and smoking condition should be considered if they are available in each individual study to obtain a more precise result. Thirdly, the gene-gene interaction which is important in developing complex diseases should also be included.

## Conclusions

In conclusion, the results from the present meta-analysis suggest that the survivin −31 G/C polymorphism might be correlated with an increased risk of CRC, indicating it may serve as a biomarker of disease progression. However, ACE I/D polymorphism is not associated with CRC risk. Further large and well-designed studies in various populations are needed to confirm our results. Moreover, the studies of gene-gene and gene-environment interactions between these polymorphisms and CRC risk should also be performed and considered.
